# Experimental and artificial intelligence molecular models to predict quenching behavior of carbon materials from petroleum waste for sustainable corrosion monitoring

**DOI:** 10.1039/d5ra02534f

**Published:** 2025-10-16

**Authors:** Maimuna U. Zarewa, Tawfik A. Saleh

**Affiliations:** a Department of Chemistry, King Fahd University of Petroleum & Minerals Dhahran 31261 Saudi Arabia tawfikas@hotmail.com tawfik@kfupm.edu.sa https://faculty.kfupm.edu.sa/chem/tawfik/publications.html

## Abstract

Quantum dots have attracted a lot of interest because of their special optical characteristics and potential for a variety of uses, such as in inks and sensors. Additionally, petroleum coke recycling is crucial for resource efficiency, economic growth, and environmental sustainability. It solves problems linked to carbon emissions and landfill waste. Industries can reduce their environmental impact while generating new opportunities for innovation and expansion. Petroleum coke quantum dots (PCQDs) were synthesized using the reflux method at 120 °C for 12 h, then characterized using scanning electron microscopy (SEM), X-ray diffraction (XRD), Fourier transform infrared spectroscopy (FTIR), and electron pair resonance spectroscopy (EPR), and were utilized to differentiate Fe^2+^ and Fe^3+^ for early-stage corrosion detection in real samples. LOD values of 0.39 µM and 0.36 µM for Fe^2+^ and Fe^3+^ were obtained using a Stern–Volmer plot. PCQDs demonstrate outstanding selectivity for iron in the presence of diverse cationic and anionic interferents, as well as remarkable stability under harsh continuous optical and thermal conditions. In addition, due to the intense blue emission of PCQDs, they have been utilized as a fluorescent security invisible ink for documenting sensitive information. The PCQDs exhibit around 70 wt% yield, 50% quantum yield, and a half-life of 9.5 ns. Due to their excellent efficiency and simplicity in synthesis, PCQDs can be utilized for industrial-scale production.

## Abstract 2

Quantum dots have attracted a lot of interest because of their special optical characteristics and potential for a variety of uses, such as in inks and sensors.

## Graphical abstract

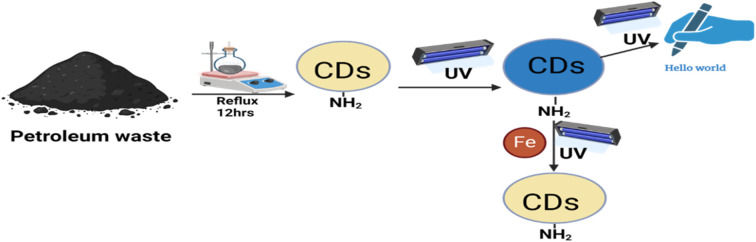

## 1. Introduction

Solid petroleum coke (PC) is a solid byproduct of oil refining that is prized for its low cost and high calorific value, and its production has increased along with processed heavy oil consumption. It is popular in the industrial sector due to its accessibility and low cost.^1^ Petroleum coke, a cheap substitute for coal in local industry, is a waste product of oil refineries. It is classified as dark solid carbon. Its use has been questioned as it emits 5–10% more CO_2_ than coal, contributing to higher pollution levels.^2^ Consequently, there is a growing need to convert this material into value-added carbon products in an eco-friendly manner. PC, with its high carbon content and a low ash level, serves as an excellent precursor for the synthesis of porous carbons.^3^ The abundance of aromatic domains, or benzene rings, in petroleum coke enables the formation of high-quality carbon quantum dots (CQDs).^4^

High-quality carbon quantum dots can be formed using a variety of techniques, which can be broadly categorized as “top-down”,^5,6^ and “bottom-up”.^7^ Bottom-to-up methods involve building molecules into quantum dots *via* self-assembly or chemical reaction. They can be obtained using many approaches, such as the soft-template method,^8–10^ hydrothermal method,^11–13^ microwave-assisted hydrothermal method,^14–16^ metal-catalyzed method,^17^ and electrosynthesis.^18^ Top-to-down methods involve breaking down bulk precursors into quantum dots. They can be subdivided into the liquid exfoliation method,^19–21^ and the electron beam lithography method.^22^ Carbon quantum dots are commonly used in a variety of applications, like light-emitting devices,^23,24^ solar cells,^25,26^ photovoltaic devices,^27^ bioimaging,^28–30^ sensing,^31,32^ catalysis,^33,34^ supercapacitors,^35,36^ nanomedicine^37^ and security invisible inks.^38^

The interesting applications of carbon quantum dots are due to their interesting properties: their functionalized surfaces show an amazing capacity to interact with ions or chemical compounds in solution selectively and sensitively, resulting in optical reactions like quenching or fluorescence amplification. Examples of fluorescence responses used in nano sensors for different anions^39^ and metal ions,^40,41^ as well as for specific chemical compounds^42^ and pH^43^ levels, may be found in the literature. Targeted sensing and detection of a wide range of analytes, such as anions, metal ions, metal nanoparticles, biological samples, and organic molecules, can be achieved through the engineering of CQDs. By altering the surface functional groups, one may fine-tune this nano-sensing ability, which in turn affects the optical properties of the CQDs when they interact with the analytes.^44^

Darani and his team in 2024 presented a novel method for metal corrosion prevention by adding carbon dots to electrodeposited coatings. To identify corrosion, CQDs were manufactured and analyzed using FTIR, SEM, photoluminescence spectroscopy, and fluorescence microscopy. Design of experiments (DOE) was used to determine the optimal parameters, resulting in improved adhesion and a 10% increase in corrosion resistance: 0.4 wt% CQD, 165 °C curing, and 36.2 V. Because CQD photoluminescence enabled real-time, non-destructive corrosion monitoring *via* UV-induced quenching, this method shows promise for industrial applications by lowering maintenance costs and increasing equipment life.^45^

Liao and his group in 2024 introduced phytic acid carbon dots (PA-CDs), a green “off–on” fluorescent sensor, in a sustainable and effective corrosion detection method. By sensing OH^−^ and H^+^ levels, PA-CDs can detect corrosion; when OH^−^ rises, fluorescence turns off, and when H^+^ is introduced, it turns on. The predicted detection limits for OH^−^ and H^+^ were 7.3 µmol L^−1^ and 20.34 µmol L^−1^. Because of deprotonation and protonation, PA-CDs aggregate and disaggregate, causing an “off–on” phenomenon. PA-CDs demonstrated their potential for useful corrosion monitoring applications by visibly indicating early-stage corrosion by a fluorescence “turn-off” effect when employed as a film on Q235 steel.^46^

Quantum dots are also utilized as invisible fluorescent security ink, and Gopi and his team in 2024 addressed the demand for innovative anti-counterfeiting materials because conventional techniques such as QR codes, holography, and barcodes are readily copied. The luminous characteristics of carbon dots (CDs) make them a suitable solution. The study outlines a quick, inexpensive, one-step method for creating CDs that produce blue light. These CDs were used to create an invisible marker by integrating them into a commercial sketch pen. Under normal light, writing created with this marker is invisible; nonetheless, when exposed to 365 nm UV light, its fluorescence is blue. The six-month stability of the fluorescence showed that CDs have promise for use in anti-counterfeiting applications.^47^

In this work, a highly selective on and off quantum dot (PCQD) fluorescent sensor derived from solid petroleum waste was utilized for the detection of Fe^3+^/Fe^2+^ for efficient early-stage corrosion monitoring. The prepared PCQDs were also evaluated as invisible fluorescent ink. They showed excellent optical performance in both applications.

## 2. Experimental

### 2.1. Materials

All the materials procured from Sigma-Aldrich (now MilliporeSigma) were used without further purification. Ultrapure water had been processed using the Milli-Q system (Milford, Massachusetts, USA). Metal ion precursors in the form of nitrates (Cr^3+^, Cr^6+^, Mg^2+^, K^+^, Pb^2+^), acetates (Na^+^, Ca^2+^, Ba^2+^, Fe^3+^, Fe^2+^, Zn^2+^, Cu^2+^, Co^2+^, Ni^2+^, Mn^2+^, Ce^2+^, Al^3+^), or chlorides (Li^+^, Cd^2+^, V^5+^, Hg^2+^, Pb^2+^) were used as well as different potassium salts (F^−^, Cl^−^, Br^−^, I^−^, NO_3_^−^, CH_3_COO^−^, SO_4_^2−^, SO_3_^2−^, and CO_3_^2−^) as anion precursors.

### 2.2. Synthesis

Solid petroleum coke (PC) with elemental composition C 65.5%, N 9.6%, H 2.2%, S 1.7% was obtained from Ras Tanura. To obtain a uniformly sized precursor, PC was crushed and passed through a 2 mm sieve. An ethanol–water mixture (6 : 4) was combined with a 1 : 1 wt ratio of petroleum coke and urea. The mixture was sonicated (DR-P60) for 15 min, followed by refluxing at 120 °C and 350 rpm for 12 h. The resulting mixture was filtered using a 0.2 µm filter paper. The filtrate was collected, and its fluorescence emission was observed under UV light before lyophilization. The resulting yellowish powder (petroleum coke quantum dots) was stored in an airtight container for further testing and characterization, with a yield of 70 wt% based on the starting petroleum coke and a quantum yield of 50% using rhodamine B as a reference. All measurements were conducted without further purification at 0.1 mg mL^−1^ of carbon quantum dots. The fluorescence quantum yield was calculated using eqn (1):1
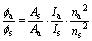
where the subscripts ‘a’ and ‘s’ denote the unknown sample and the fluorescence reference standard, respectively. In turn, *A*, *I* and *n* represent the absorbance, the integrated emission spectrum, and the refractive index, respectively.^48^

### 2.3. Characterization

**Scheme 1:**
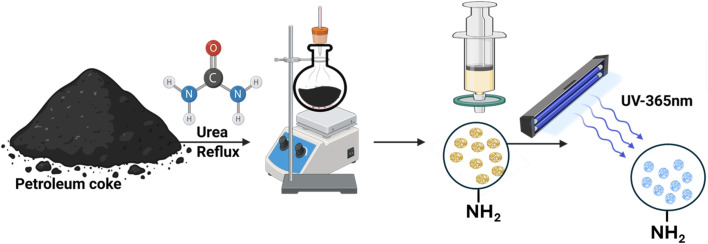
Schematic illustration of the preparation steps.

X-ray photoelectron spectroscopy (XPS) analysis was conducted using a Thermo Scientific Escalab 250Xi spectrometer equipped with an Al Kα (1486.6 eV) X-ray source, operating at an energy resolution of 0.5 eV. High-resolution scans used a pass energy of 30 eV, while survey scans used a 650 µm beam and a pass energy of 100 eV. A Quattro FESEM 400 high-resolution field emission scanning electron microscope (SEM) at 20 keV was employed for morphology, EPR, and attenuated reflectance investigations. Using a PerkinElmer 16F PC FTIR spectrometer, Fourier transform infrared (ATR-FT-IR) measurements were performed, and XRD was performed with a Rigaku Ultima IV X-ray diffractometer with a 5–70° 2*θ* scan range at a 3° min^−1^ speed, 40 kV, and 40 mA. UV-visible spectra were obtained using a Thermo Scientific spectrophotometer in the range of 200–800 nm. For repeatable runs, the sealed capillary tube was placed inside an EPR resonator that had been previously adjusted and fitted with a 120 mm (length) × 3 mm (OD) EPR tube. Modulation amplitude = 0.010 mT (0.1 G), microwave power (100 mW), and CW X-band operation were used with an EPR benchtop SPINSCAN-X spectrometer (ADANI, Minsk, BLR). A Hamamatsu Quantaurus-Tau fluorescence lifetime spectrometer was used to record time-dependent PL data and room temperature steady-state PL measurements, and a Genesys 10S to scan the UV absorbance of the PCQDs. A quartz cuvette with a width of 10 mm was used to record all the spectra under ambient conditions for both PCQD solutions and actual samples for corrosion testing (Scheme 1).

## 3. Results and discussion

### 3.1. Characterization

**Fig. 1:**
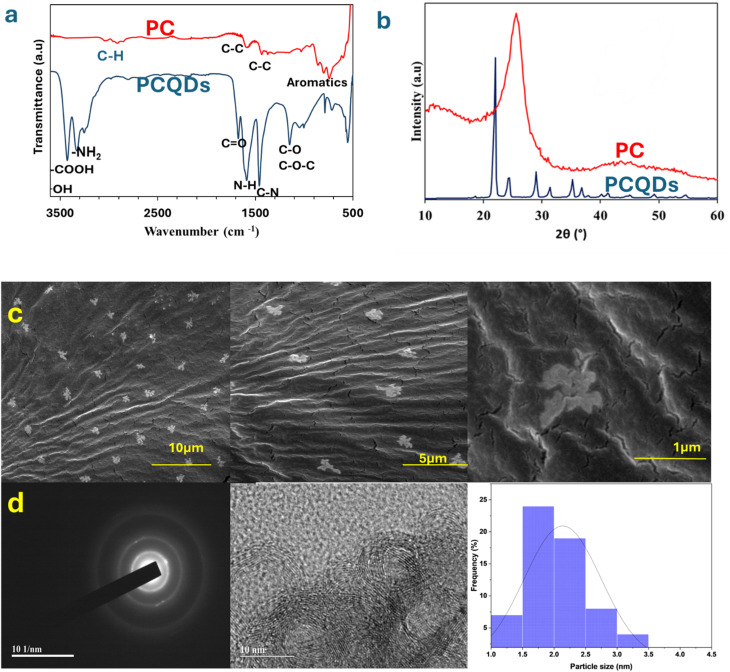
(a) FTIR spectra of PCQDs and PC. (b) XRD spectra of PCQDs and PC. (c) SEM images of PCQDs. (d) SAED, TEM and particle size distribution curve of PCQDs.

**Fig. 2:**
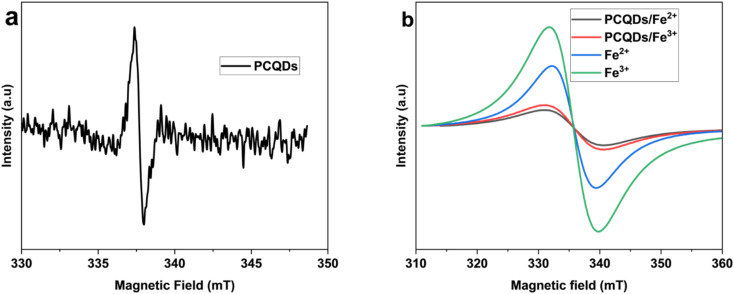
(a) EPR spectrum of PCQDs at room temperature. (b) Comparison of EPR spectra of PCQDs with Fe^2+^ and Fe^3+^ at room temperature.

PCQDs were obtained by refluxing PC for 12 h and were then characterized using a range of techniques, such as XRD, SEM, and FTIR. The XRD pattern of the PC showed a (002) plane at 25.6° and a (101) plane at 44°, revealing the graphitic nature of the PC. The observed change in the XRD pattern of the PCQDs is due to the modifications and functionalization of the PC with urea, which resulted in a more crystalline and ordered structure (Fig. 1b). The chemical structure and surface functionalities of the PCQDs were examined using FTIR (Fig. 1a). The C=O stretching vibration and the C–N vibration were observed at 1674 cm^−1^ and 1458 cm^−1^. Vibration linked to the N–H group was observed at 1587 cm^−1^, whereas other peaks verify that the PCQD surface contains hydroxyl, amide, and carboxyl (3190 cm^−1^) groups and amino NH (stretching at 2930 cm^−1^). A peak at 1147 cm^−1^ corresponds to C–O and C–O–C functionalities.^49,50^ Moreover, FTIR showed C–H, C–C, and aromatics peaks at 2914 cm^−1^, 1587 cm^−1^, and 799 cm^−1^.^51^ A high increase in nitrogen content in the elemental analysis of the PCQDs from 9.6% to 35% was observed with C/N ratios of 6.8 and 0.5 before and after nitrogen doping, showing a significant decrease in C and an increase in N. This proves the successful functionalization on the surface of the PCQDs with a nitrogen group because of treatment with urea. The average particle size was assessed to be 2.1 ± 0.6 nm using TEM with 64 particles sampled (Fig. 1d). The SEM image in Fig. 1c reveals the distinctive bird-like form of the PCQDs. Because of their unique structure, PCQDs offer good sensitivity and enhanced selectivity for sensing Fe ions. The EPR spectra of the PCQDs in DMF at 5 mg mL^−1^ at room temperature are displayed in Fig. 2. Due to the high level of functionalization with the nitrogen group, the PCQDs act as an electron donor. The electron donor behavior of the PCQDs is revealed by their *g*-factor of 1.9992. Fe^2+^ and Fe^3+^ were observed to exhibit different behaviors in relation to the PCQDs. Fe^2+^ demonstrated a greater coupling effect and stronger interaction between PCQDs and Fe^2+^, with an increase in *g*-factor from 2.0094 for Fe^2+^ to 2.0098 for PCQDs-Fe^2+^, and Fe^3+^ showed a slightly weaker interaction with a *g*-factor from 2.0096 for Fe^3+^ to 2.0085 for PCQDs-Fe^3+^. Given that the fluorescence intensity decreased more with Fe^2+^ and Fe^3+^, these results are consistent with the PL findings. Because of N-doping, the PCQDs act as an electron donor in both scenarios, while the metals act as electron acceptors with a stronger affinity of PCQDs toward Fe^2+^. This behavior led to the quenching of the fluorescence and sensing of Fe^2+^ and Fe^3+^.

**Fig. 3:**
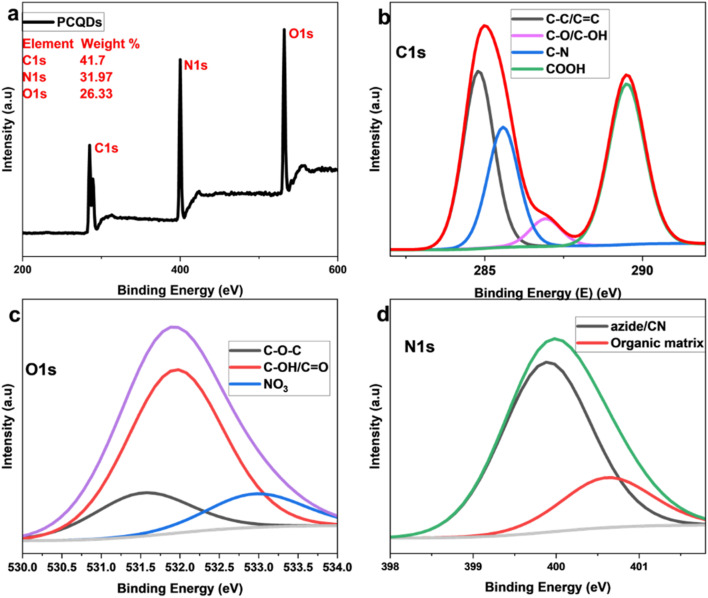
(a) XPS survey spectrum of PCQDs. (b) C 1s XPS deconvolution. (c) O 1s XPS deconvolution. (d) N 1s XPS deconvolution.

X-ray photoelectron spectroscopy (XPS) elemental analysis recorded 41.7% carbon, 31.97% nitrogen, and 26.33% oxygen, confirming the successful functionalization of the PCQDs with the nitrogen group (Fig. 3). XPS examination of nitrogen-doped PCQDs reveals complicated surface chemistry that enables the selective identification of Fe^2+^ and Fe^3+^. The distinct interaction of functional groups added during doping and subsequent oxidation seems to be the driving force for this selectivity. Nitrogen has successfully assimilated into the carbon framework, as evidenced by the C–N bonds found in the carbon analysis. XPS confirms nitrogen-containing groups (C–N) and oxygenated groups (C–O, C=O) on the PCQDs. These sites act as Lewis bases and provide coordination points for Fe^2+^/Fe^3+^. Consistent with our EPR data, the nitrogen sites donate electron density and facilitate interfacial electron transfer during sensing. Carbonyl groups (C=O) can coordinate Fe *via* lone pairs and, together with C–O, improve hydrophilicity and dispersion in water. The combined presence of nitrogen and oxygen functionalities creates a heterogeneous, polar surface that promotes Fe binding and stabilizes Fe–PCQD interactions. This cooperative effect explains the strong response and selectivity towards Fe^2+^/Fe^3+^ ions.

Proton NMR showed more sp^2^ carbon than sp^3^ carbon, and the dominance of sp^2^ carbon was confirmed by C-13 NMR,^52^ suggesting a graphitic conjugated system that favors fluorescence emission and sensing (SI Fig. S1).

**Fig. 4:**
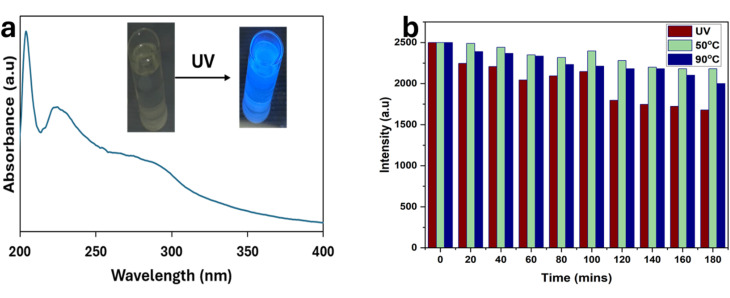
(a) UV-visible spectroscopy of PCQDs. Inset: PCQDs under daylight and 365 nm UV light. (b) Photo and thermal stability test of PCQDs for 3 h under UV light, at 50 °C, and at 90 °C.

**Fig. 5:**
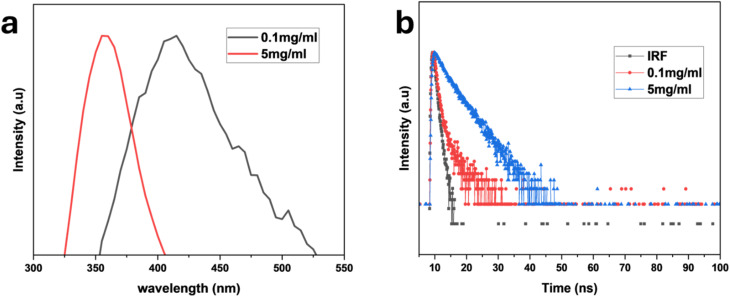
(a) Emission spectra of PCQDs at various concentrations. (b) Time-resolved decay profiles of PCQDs at various concentrations.

The optical properties of the PCQDs were analyzed using UV-vis and PL (Fig. 4). To obtain the maximum PL intensity, UV-vis analysis was conducted on PCQDs from 199 nm to 500 nm. The absorbance between 220 and 300 nm is assigned to π–π* and n–π* transitions associated with the C–O, C–N, and C=O functional groups (Fig. 4a).^53^ A high quantum yield of about 50% can be linked to the role of nitrogen doping in the PCQDs, which created a modified and reorganized surface state that led to a complex π hetero-conjugation that intensified the radiation.^54,55^ The emission spectra of the PCQDs are concentration-dependent; a blue shift was observed at a higher concentration (5 mg mL^−1^). This is due to the aggregation of the PCQDs, which produces different optical and electrical environments. An overall shift in emission towards shorter wavelengths results from proximity, which modifies the way energy is transported within the aggregate and causes loss of energy by non-radiative means. At the same time, reabsorption takes center stage in concentrated PCQD solutions. A nearby PCQD absorbs photons released by another PCQD in this process, preventing them from leaving the solution. Red light, which has a longer wavelength, is more likely to be reabsorbed than blue light, which has a shorter wavelength. Shorter wavelengths are therefore more likely to escape without additional absorption, but longer wavelengths are selectively reabsorbed. Longer-wavelength emissions appear less intense due to this filtering effect, which moves the emission spectrum toward the blue end. The blue shift seen in concentrated PCQD solutions can be explained by both aggregation and reabsorption. Aggregation alters the electronic states and decreases brightness, whilst reabsorption preferentially allows shorter wavelengths to escape. The significance of regulating the PCQD concentration is shown by these combined effects (Fig. 5a). The decay dynamics at 280 nm excitation and 300 nm emission could be fitted to a nonlinear exponential decay curve, and the half-life of the PCQDs is concentration dependent: at 0.1 mg mL^−1^, it was 9.5 ns, and at 5 mg mL^−1^, it dropped to 7.4 ns. This is because higher concentrations of PCQDs may cause them to aggregate or self-quench, in which non-radiative energy transmission between the particles occurs due to their proximity. As more excitons recombine non-radiatively instead of releasing photons, this mechanism may shorten the half-life of the photoluminescence (Fig. 5b).

**Fig. 6:**
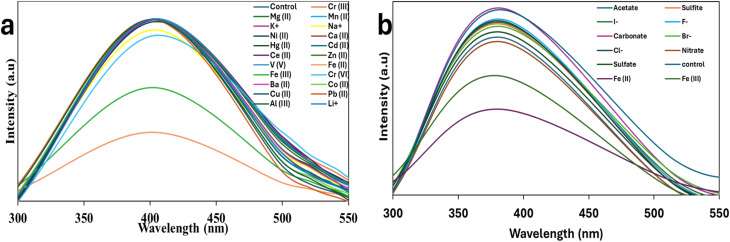
(a) Photoluminescence spectra showing different metal ion selectivity and (b) anion selectivity.

Thermal stability tests were conducted at 50 °C and 90 °C continuously for 3 h, and the PCQDs showed excellent thermal stability even at 90 °C. Additionally, the photostability of the PCQDs was tested by continuously irradiating them with UV light at 365 nm for 3 h, and these tests were monitored with PL at a 280 nm excitation wavelength. Evaluating the sensitivity of the PCQDs to different ions was crucial because of their intrinsic luminous characteristics and photo and thermal stability for 3 h. Subsequent analysis showed that the produced PCQDs were sensitive to iron species that are soluble in water, specifically Fe^2+^ and Fe^3+^. Health problems can arise from either an excess (hyperferremia) or a deficit (hypoferremia) of Fe^2+^, an important bioactive transition metal ion. Additionally, Fe^2+^ and Fe^3+^ are important for biological activities and have a big impact on corrosion processes, which affects the integrity of metals in different situations. PCQDs can therefore play a vital role in the detection of early corrosion processes. At concentrations of 50 ppm in aqueous solutions, the PCQDs showed remarkable selectivity towards a variety of environmentally relevant cations, including Ba^2+^, Pb^2+^, Cr^6+^, Cu^2+^, Na^+^, K^+^, Ca^2+^, Mg^2+^, V^5+^, Al^3+^, Hg^2+^, Cd^2+^ Co^2+^, Ni^2+^, Cu^2+^, Zn^2+^, Cr^3+^, Mn^2+^, and Li^+^, as well as anions such as F^−^, Cl^−^, Br^−^, I^−^, NO_3_^−^, SO_4_^2−^, SO_3_^2−^, CH_3_COO^−^, and CO_3_^2−^ (Fig. 6). For precision and dependability, every experiment was run in triplicate, especially the ones that targeted Fe^2+^ and Fe^3+^. The PCQDs-Fe^2+^/Fe^3+^ emissions showed no observable redshift, and this suggests direct interaction between the PCQDs and Fe^2+^/Fe^3+^.

### 3.2. Early corrosion monitoring

**Fig. 7:**
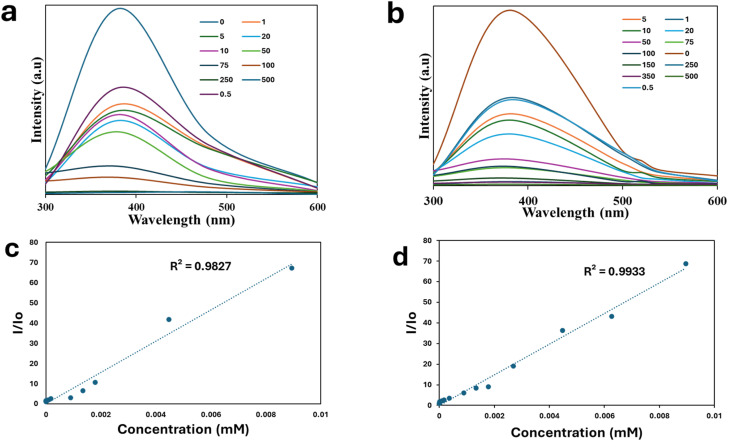
(a) Photoluminescence spectra of various Fe^2+^ concentrations. (b) Photoluminescence spectra of different concentrations of Fe^3+^. Stern–Volmer plot for (c) Fe^2+^, and (d) Fe^3+^.

We further studied the sensitivity of the PCQD fluorescent probe towards Fe^2+^ and Fe^3+^ to determine the limits of quantification (LOQ) and detection (LOD). The fluorescence of the PCQDs at 280 nm is quenched by adding Fe^2+^ and Fe^3+^. This led to a decrease in emission intensity that showed a linear relationship with increasing Fe^2+^ and Fe^3+^ concentrations (Fig. 7a and b). Interestingly, there was no change in the maximum of the fluorescence emission. For Fe^2+^ (*R*^2^ = 0.9827) and Fe^3+^ (*R*^2^ = 0.9933), the Stern–Volmer plots displayed an excellent correlation. This resulted in quenching constant values of *K*_sv_ = 7734 (found by analyzing the line slope as *y* = 7734*x*) and of Ksv = 7421.6 (found by analyzing the slope as *y* = 7421.6*x*) for Fe^2+^ and Fe^3+^. The limit of detection (LOD) was estimated by the formula LOD = 3*σ*/*K*_sv_, while the limit of quantification (LOQ) was determined with the equation LOQ = 10*σ*/*K*_sv_, where *σ* represents the standard error of the intercept from the Stern–Volmer plots. For Fe^3+^, a LOD of 0.36 µM and a LOQ of 1.19 µM were obtained (*σ* = 0.88422). In contrast, for Fe^2+^, the LOD was found to be 0.39 µM with a LOQ of 1.29 µM (*σ* = 0.99597).^56^

Corrosion monitoring was evaluated using real samples of water. The sample was centrifuged and spiked separately with Fe^2+^ and Fe^3+^, and the PL measurements were conducted after 5 min (Fig. S2). By utilizing PCQDs as a fluorescence-based sensor, the suggested method enables accurate, real-time corrosion monitoring through the selective detection of Fe^2+^ and Fe^3+^ ions. PCQDs are very useful for this application because of their special optical characteristics, which include intense fluorescence and selective response to various iron oxidation states. Through monitoring variations in fluorescence intensity upon detection of Fe^2+^ and Fe^3+^ ions, the PCQD sensor will enable ongoing evaluation of corrosion activity. Table 1 shows data demonstrating near-100% recovery rates and low relative standard deviation (RSD) values for both Fe^2+^ and Fe^3+^ species in water samples, highlighting the great sensitivity and accuracy of the PCQDs,^57^ which offer substantial advantages in corrosion detection. As these species are markers of various phases of material degradation, the ability to differentiate between Fe^2+^ and Fe^3+^ concentrations (Fig. 7) offers important insight into the corrosion processes (Table 1). The long-term integrity and durability of materials exposed to corrosive environments will be enhanced by the early detection of corrosion onset, the timely application of preventive measures, and the integration of this quantum dot fluorescent sensor into a corrosion monitoring framework. The reported materials show excellent performance as fluorescent probes for Fe^2+^ and Fe^3+^ detection compared to several materials reported in the literature, as shown in Table 2.

**Table 1:** Real sample detection of Fe^2+^ and Fe^3+^ using PCQDs

Sample	Analyte	Concentration added (ppm)	Concentration found (ppm)	RSD	% Recovery	Fe^3+^/Fe^2+^
Drinking water	Fe^2+^	5	4.974 ± 0.058	1.167	100.523	0.961
	Fe^3+^	5	4.784 ± 0.057	1.193	104.499	
Tap water	Fe^2+^	5	5.064 ± 0.103	2.035	98.721	1.091
	Fe^3+^	5	4.640 ± 0.062	0.013	107.750	

**Table 2:** Comparison of different quantum dot fluorescent probes for Fe^2+^ and Fe^3+^ detection

Quantum dots	Analyte	LOD (nM)	Quantum yield (%)	pH	Ref.
NCQDs	Fe^3+^	0.21	55	Acidic	58
NCDs	Fe^3+^	800	15.13	Neutral	59
Cdots	Hg^2+^ and Fe^3+^	75	50.78	Neutral	60
N,S-CDs	Fe^3+^	200	20.5	Basic	61
CNPs	Fe^3+^	1060	5.42	Neutral	62
C-QDs	Fe^3+^	1300	50	Neutral	63
mPD-CDs	Fe^2+^	590	74.13	Acidic	64
N,Cl-CDs	Fe^2+^	300	15.2	Neutral	65
NR-CDs	Fe^2+^	14	21	Neutral	66
PCQDs	Fe^2+^ and Fe^3+^	390 and 360	50	Neutral	This work

### 3.3. Fluorescent invisible ink

**Fig. 8:**
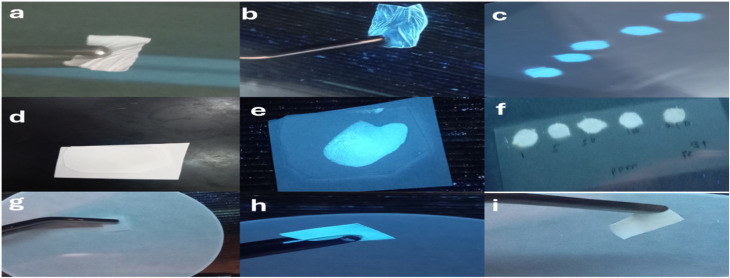
(a) and (b) PVDF membrane before and after PCQD coating under UV-365 nm. (d) and (e) Invisible fingerprint under normal daylight and under UV-365 nm. (g)–(i) Filter paper before PCQD coating (UV-365 nm), after PCQD coating under UV-365 nm, and after adding 75 ppm of Fe^3+^ (UV-365 nm). (c) and (f) Filter paper coated with PCQDs and after adding Fe^3+^ at various concentrations under UV-365 nm.

It is imperative to extend the use of emerging PCQDs into novel applications. The PCQDs produced in this study have several excellent, noteworthy characteristics, such as remarkable thermal and photostability, a high quantum yield of 50%, and good transparency in the visible spectrum. The PCQDs can be used as invisible ink because of these qualities. Information was written on commercially available filter paper^67^ with the PCQD concentration in the ink kept at 6 mg mL^−1^. Words written on filter paper with the probe appeared only under a 365 nm UV lamp and were invisible in daylight (Fig. S3 and S4). On a similar note, Fig. 8d and e reveal fingerprints that are invisible under normal daylight. Because of the PCQDs' exceptional stability, this visibility is reliable and consistent in ambient settings. Consequently, we suggest that this invisible ink based on PCQDs can efficiently transmit sensitive and crucial security information for safe communications.^68^ Furthermore, the PCQD/PVA composite film exhibits intense blue fluorescence under UV light upon combination with PVA (Fig. 8a and b). On a similar note, filter paper was observed before and after coating with PCQD ink, and it exhibits blue fluorescence (Fig. 8g and h, respectively). A micro-drop of Fe^2+^ was added to the coated paper and the blue fluorescence disappeared. This phenomenon can be utilized in the development of a test paper for preliminary Fe^2+^/Fe^3+^ sensing, as shown in Fig. 8c, f and i.

### 3.4. Pretrained molecular models for quantum dot–quencher interaction prediction

**Fig. 9:**
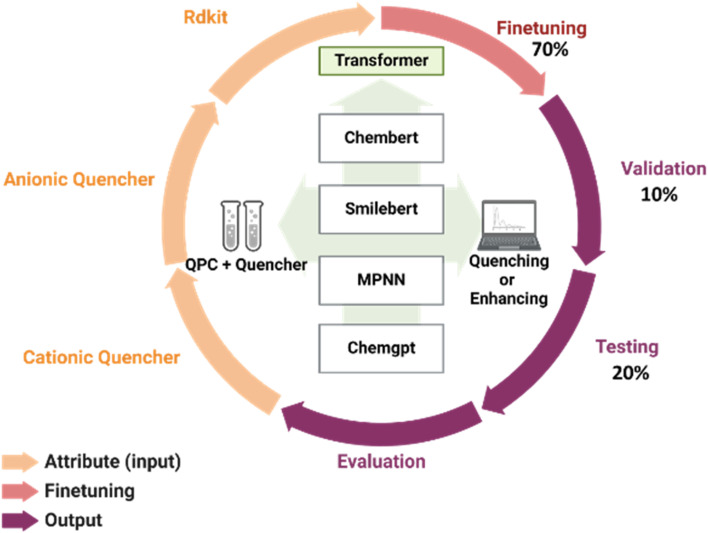
Schematic illustration of Q and E prediction workflow.

The experimental findings of the selectivity analysis employing PCQD PL intensity and quencher qualities, such as SMILES and RDKit features (molecular weight, charges), to optimize and predict the quenching (Q) or enhancing (E) behaviors of quenchers are shown in this section. The process is depicted in Fig. 9. 10% of the dataset was used for validation, 20% for testing, and 70% for fine-tuning pretrained models.

The pretrained models that were assessed from Hugging Face consist of:

(i) ChemBERT, which uses transformer models to predict chemical properties, and has shown its scalability, competitiveness, and visualization abilities using a carefully selected dataset of 77 million SMILES.^69^

(ii) SMILEBERT, which is a semi-supervised BERT model for SMILES representations.^70^

(iii) MPNN, which uses a new message-passing technique on undirected graphs.^71^

(iv) ChemGPT, which emphasizes the interaction between scaling and physical priors for predictions of interatomic potentials and generative chemistry.^72^

**Fig. 10:**
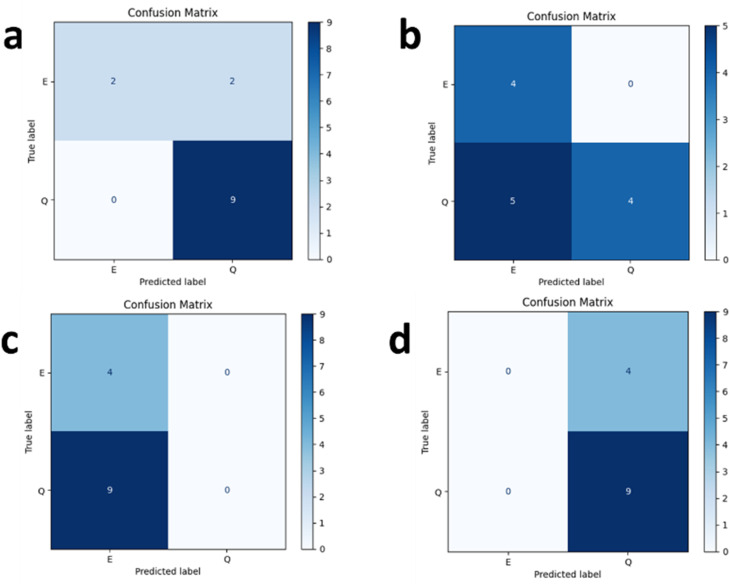
Confusion matrix of PCQD-quencher interaction using (a) ChemBERT, (b) SMILEBERT, (c) MPNN, and (d) ChemGPT.

PyTorch was used to implement all models with the following fine-tuning hyperparameters: a batch size of 8, a learning rate of 1 × 10^−5^, and 10 epochs. The mean and standard deviation of the ROC-AUC scores for the PCQD selectivity dataset are shown in Table 3. ChemBERTa's efficient scaling using pretraining data and attention-based visualization allowed it to achieve an overall accuracy of 87%, which was a substantial improvement over the other models. MPNN was 100% biased towards enhancing, whereas ChemGPT was 100% biased toward quenching, according to the confusion matrix analysis in Fig. 10. ChemBERTa proved to be more successful at identifying class distinctions, proving its usefulness for this task.

**Table 3:** The metric scores of the test set

Pretrained model	MPNN	SMILEBERT	ChemGPT	ChemBERT
Mean accuracy	0.25 ± 0.16	0.58 ± 0.13	0.75 ± 0.16	0.87 ± 0.11

## 4. Conclusion

Quantum petroleum coke (PCQDs), a nitrogen-doped substance with good and consistent luminous qualities, was created in this study by treating petroleum coke with urea. Because of this alteration, PCQD fluorescence is enhanced, which makes it perfect for “on-off” detection of Fe^2+^ and Fe^3+^, where the presence of these ions causes a change in fluorescence. The selectivity of PCQDs towards Fe^2+^ and Fe^3+^ enables them to operate as a very sensitive sensor. Besides their ability to sense ions, PCQDs exhibited remarkable selectivity among various cations and anions, ensuring dependable detection even in complex environments containing multiple interfering ions. Their strong fluorescence also renders PCQDs ideal as a security ink for applications aimed at preventing counterfeiting. Moreover, the structure of PCQDs can be altered to broaden their applicability in energy storage sectors, such as for batteries or supercapacitors. PCQDs can be fine-tuned by modifying their composition to enhance performance, demonstrating their versatility across different technologies.

## Conflicts of interest

There are no conflicts to declare.

## Supplementary Material

RA-015-D5RA02534F-s001

## Data Availability

The software packages employed can be obtained either freely or through commercial licensing from their respective providers. Other data can be provided by the authors on request. Supplementary information is available. See DOI: https://doi.org/10.1039/d5ra02534f.
