# Supplementary material for: Experimental and artificial intelligence molecular models to predict quenching behavior of carbon materials from petroleum waste for sustainable corrosion monitoring
Source: RSC Adv. 2025 Oct 16;15(46):39059–70. doi: 10.1039/d5ra02534f (PMC12530849; doi:10.1039/d5ra02534f)
Supplement: RA-015-D5RA02534F-s001 [file RA-015-D5RA02534F-s001.pdf]

Supplementary Information (SI) for RSC Advances.
This journal is © The Royal Society of Chemistry 2025

SUPPLEMENTARY INFORMATION

Experimental and Artificial Intelligence Molecular Models to Predict Quenching Behavior of Carbon materials from Petroleum Waste for Sustainable Corrosion Monitoring

Maimuna U. Zarewa [1], Tawfik A. Saleh [1*]

[1] *Department of Chemistry, King Fahd University of Petroleum & Minerals, Dhahran 31261, Saudi Arabia*

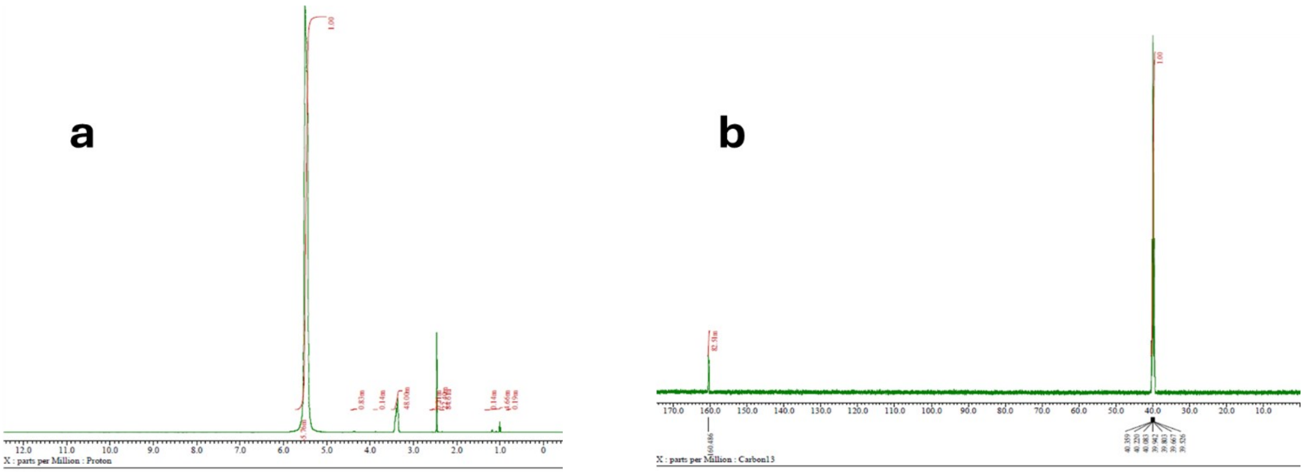

Figure S1: a) Proton NMR of QPC in DMSO b) Carbon 13 NMR of QPC in DMSO

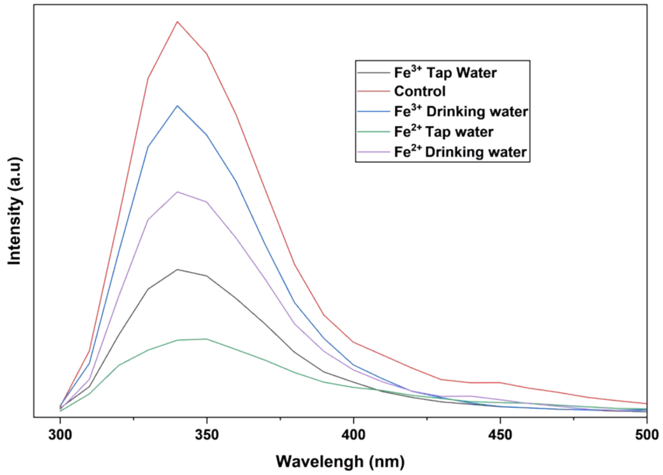

Figure S2: PL spectra of Control (QPC fluorescence sensor) in real samples of spiked Drinking water and tap water

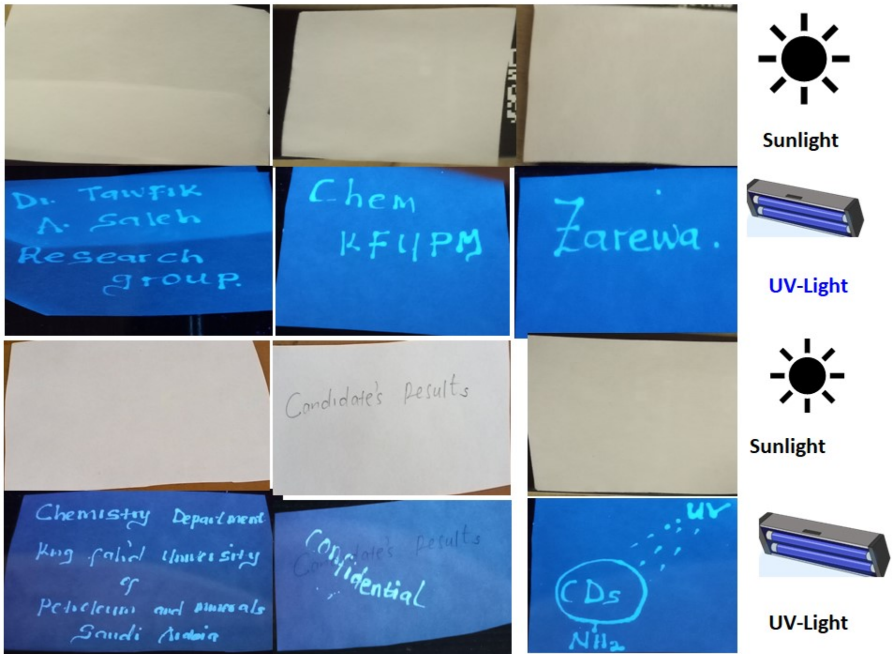

Figure S3: Various writings using invisible ink under sunlight and under UV-light.

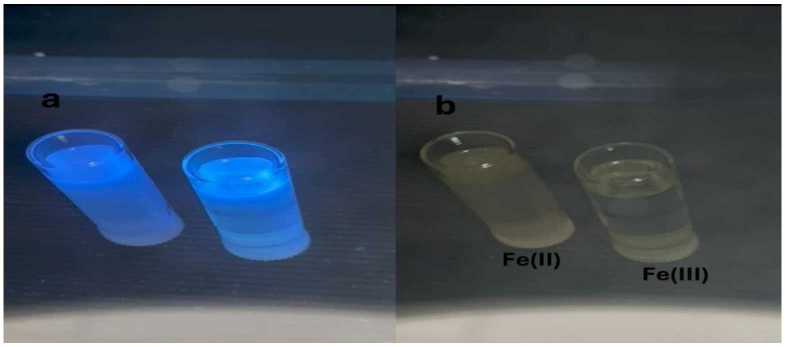

Figure S4: a) QPC under UV 365nm b) QPC with Fe(II) and Fe(III) under UV 365nm.
